# Elevated Blood Urea Nitrogen to Serum Albumin Ratio Is an Adverse Prognostic Predictor for Patients Undergoing Cardiac Surgery

**DOI:** 10.3389/fcvm.2022.888736

**Published:** 2022-05-04

**Authors:** Liu Ye, Haoming Shi, Xiaowen Wang, Qin Duan, Ping Ge, Yue Shao

**Affiliations:** ^1^The First Branch, The First Affiliated Hospital of Chongqing Medical University, Chongqing, China; ^2^Department of Cardiothoracic Surgery, The First Affiliated Hospital of Chongqing Medical University, Chongqing, China

**Keywords:** urea nitrogen, serum albumin, prognosis, intensive care unit, cardiac surgery

## Abstract

**Background:**

Elevated blood urea nitrogen (BUN) and reduced albumin have been prominently correlated with unfavorable outcomes in patients with cardiovascular diseases. However, whether combination BUN and albumin levels could predict the adverse outcomes of cardiac surgery patients remains to be confirmed. Here, we investigated the prognostic effect of the preoperative BUN to serum albumin ratio (BAR) in cardiac surgery patients.

**Methods:**

Data were obtained from the Medical Information Mart for Intensive Care (MIMIC) III and eICU databases and classified into a training cohort and validation cohort. The BAR (mg/g) was calculated by initial BUN (mg/dl)/serum albumin (g/dl). The primary outcome was in-hospital mortality. Secondary outcomes were 1-year mortality, prolonged length at intensive care unit, and duration of hospital stay. The associations of BAR with outcomes were explored by multivariate regression analysis and subgroup analyses. Then, C statistics were performed to assess the added prognostic impact of BAR beyond a baseline risk model.

**Results:**

Patients with in-hospital death had significantly higher levels of BAR. Multivariate regression analysis identified BAR, as a categorical or continuous variable, as an independent factor for adverse outcomes of cardiac surgery (all *p* < 0.05). Subgroup analyses demonstrated a significant relationship between elevated BAR and in-hospital mortality in different subclasses. The addition of BAR to a baseline model provided additional prognostic information benefits for assessing primary outcome. Results were concordant in the external validation cohort.

**Conclusions:**

Increased preoperative BAR is a potent predictor of unfavorable outcomes in patients undergoing cardiac surgery.

## Introduction

Approximately two million people undergo cardiac surgical procedures annually worldwide (1). As a population ages, the incidence of cardiovascular diseases rises, resulting in a growing number of cardiac surgeries. Despite advancements in therapeutics, reducing post-operative complications and mortality in cardiac surgery remains challenging (2, 3). Thus, identifying individuals at high risk of mortality may help risk stratification and individualized treatment.

An ideal biomarker should be easily accessible, inexpensive, non-invasive, and reflects the specific pathophysiological mechanisms of a disease (4). The blood urea nitrogen (BUN) to serum albumin ratio (BAR) is an emerging biomarker and was demonstrated to be linked with lung cancer and even COVID-19 (5, 6). The prognostic significance of blood urea nitrogen has been demonstrated in various cardiovascular diseases, including acute coronary syndromes (ACS), acute myocardial infarction (AMI), heart failure (HF) (7–9). The relationship between hypoalbuminemia and adverse outcome in patients after coronary artery bypass graft (CABG) and valve surgeries is well-established (10–12). Elevated BUN levels have been associated with low cardiac output and activation of the renin-angiotensin-aldosterone system (RAAS) (13, 14). Further, there is strong evidence showing the various physiological properties, including the antioxidant and anti-inflammatory effects of albumin (15–17). Thus, BUN and albumin have shown promising prognostic potential in cardiovascular disease and might be suitable biomarkers for predicting the outcomes of patients undergoing cardiac surgery. However, since the impact of BAR among cardiac surgery patients has not yet been investigated, in this study, we evaluated its prognostic ability in predicting the poor prognosis in cardiac surgery patients.

## Materials and Methods

### Study Design

The data were retrieved from two large publicly electronic databases, the Medical Information Mart for Intensive Care III version 1.4 (MIMIC III v 1.4) database and eICU Collaborative Research Database v2.0. The MIMIC III is an ICU database from the Beth Israel Deaconess Medical Center containing 58,976 ICU admissions between 2001 and 2012 (18). The eICU is a multi-center ICU database containing over 200,000 ICU admissions between 2014 and 2015 from 208 hospitals in the United States (19). We received permission to access these two databases upon completing the National Institutes of Health's web-based course and passing the Protecting Human Research Participants exam. Informed consent was waived for this study since the data were anonymously recorded. Adult patients who received cardiac surgery and were admitted to the ICU for the first time were included, while patients missing serum albumin and BUN data and patients with malignancy or liver cirrhosis were excluded from the study.

### Data Extraction

The following parameters were retrieved for each patient: (i) demographic data, including age, gender and body mass index (BMI); (ii) vital signs, including heart rate and blood pressure (BP); (iii) concomitant diseases, including hypertension, diabetes, coronary heart disease (CHD), valve disease, HF, atrial fibrillation (AF) and chronic kidney disease (CKD); (iv) vasoactive drug use, including dopamine, dobutamine, epinephrine, and norepinephrine; (v) first Sequential Organ Failure Assessment (SOFA) score; (vi) laboratory values at admission, including white blood cell (WBC) count, platelet (PLT) count, hemoglobin (Hb), BUN, albumin, serum creatine (SCr), sodium, potassium, glucose, anion gap, bicarbonate, chloride, alanine aminotransferase (ALT), and aspartate aminotransferase (AST). BAR (mg/g) was calculated from the quotient between BUN (mg/dL) and albumin (g/dL). The preoperative laboratory metrics were extracted from the first measurement recorded after admission. Cardiac surgical procedures included isolated CABG, isolated heart valve surgery, and combined surgery (CABG and valve). The primary outcome of this study was the incidence of in-hospital mortality. The secondary outcomes were incidence of ICU and 1-year death, prolonged length of ICU stay, and duration of hospital stay. Prolonged length of stay (LOS) was defined as ICU and hospitalization length of stay greater than the 75th percentile. Increased in ICU and hospitalization LOS were defined as length of stay extending to 5 and 15 days, respectively.

### Statistical Analysis

Data from the MIMIC III database was regarded as the training cohort and eICU as the external validation cohort. For extreme values, we set variables with values below the first percentile to the first percentile value and values greater than the 99th percentile to the 99th percentile value. All variables had <5% missing data and these values were replaced by their medians. Continuous data were described as median due to non-normal data distribution, and differences were compared by the Mann-Whitney U-test. The Pearson χ2 tests were employed for categorical variables, shown as counts (percentage). We constructed receiver operating characteristic (ROC) curve to determine the best cutoff value of BAR for predicting in-hospital mortality, then divided patients into two groups on the basis of the best cutoff value of BAR. Kaplan-Meier curves were applied to assess the differences between the two groups in 1-year survival rate. Multivariate logistic regression analysis and Cox proportional hazards regression analysis were applied to evaluate the influence of BAR on poor prognosis. BAR was analyzed both as a continuous and a categorical variable. Variables that *p* < 0.05 in univariate analysis in MIMIC III were used for multivariate regression analysis to exclude confounding factors. For both databases, we adjusted for age, SBP, heart rate, hypertension, HF, CKD, WBC, hemoglobin, SCr, sodium, glucose, anion gap, bicarbonate, ALT, AST, SOFA score and vasoactive in the multivariable models (Supplementary Table 2). We further performed stratified analyses to check if the prognostic effect of BAR on the primary endpoint was consistent for the different subgroups classified by gender, age, hypertension, diabetes, CHD, CKD, HF, valve disease, AF, vasoactive drug use, CABG and valve surgery. We calculated the C statistics, continuous net reclassification index (NRI) and integrated discrimination improvement (IDI) to assess the added prognostic impact of BAR beyond a baseline model, which included variables that were significant in multivariate logistic regression analysis. Additionally, we constructed ROC curve for each model and compared their area under the curve (AUC) using DeLong's test. All analyses were done using the R software (version 3.6.3) and MedCalc (version 19.1; MedCalc Software, Belgium). A *p* < 0.05 was regarded as statistically significant.

## Results

### Baseline Characteristics of the Study Population

The study comprised 2,527 and 3,138 patients from the MIMIC-III and eICU databases. Some degree of heterogeneity between the two datasets was observed. The patients' characteristics are outlined in Supplementary Table 1. The study population in each dataset was classified into two groups based on the occurrence of in-hospital mortality. All patient features are listed in Table 1. Overall, patients with in-hospital mortality had significantly higher levels of BUN and BAR, but lower level of serum albumin compared to those without. Patients who died during hospitalization in the MIMIC III had faster heart rate, were more elderly, had higher levels of WBC, SCr, anion gap, ALT, AST, and SOFA score, higher prevalence of HF, and used more vasoactive drugs. Also, patients with in-hospital death in the MIMIC-III dataset had lower levels of SBP, DBP, Hb, sodium, and bicarbonate, and lower prevalence of hypertension. In the eICU dataset, the in-hospital mortality group had higher proportions of valve disease, AF, HF and CKD, were more elderly, had increased heart rate, SCr, anion gap, ALT, AST, and SOFA score, and used more vasoactive drugs, but had decreased levels of PLT, Hb and bicarbonate, and a lower proportion of male patients (Table 1).

**Table 1:** Baseline clinical characteristics of patients based on in-hospital mortality.

**Variables**	**MIMIC III**			**eICU**		
	**Survivors**	**Non-survivors**	***P***	**Survivors**	**Non-survivors**	***P***
	***N* = 2,452**	***N* = 75**		***N* = 3,021**	***N* = 117**	
Age, years	68.7 (60.0, 77.1)	73.9 (65.8, 79.4)	0.005	68.0 (60.0, 76.0)	71.0 (63.0, 78.0)	0.031
Male, *n* (%)	1,648 (67.2%)	49 (65.3%)	0.829	2,091 (69.2%)	66 (56.4%)	0.005
BMI, kg/m^2^	27.9 (24.6, 31.6)	28.7 (25.3, 32.7)	0.158	28.8 (25.3, 33.1)	30.2 (24.2, 34.1)	0.266
SBP, mmHg	115 (104, 129)	109 (95, 127)	0.020	120 (106, 138)	116 (103, 131)	0.122
DBP, mmHg	59.0 (52.0, 67.0)	56.0 (46.5, 62.0)	0.003	65.0 (56.0, 76.0)	63.0 (52.5, 72.5)	0.100
Heart rate, bpm	84.0 (77.0, 90.0)	88.0 (78.0, 98.0)	0.006	80.0 (70.0, 91.0)	87.0 (76.3, 100.0)	<0.001
Hypertension, *n* (%)	1,469 (59.9%)	26 (34.7%)	<0.001	628 (20.8%)	23 (19.7%)	0.858
Diabetes, *n* (%)	844 (34.4%)	25 (33.3%)	0.943	521 (17.2%)	26 (22.2%)	0.205
CHD, *n* (%)	1,986 (81.0%)	54 (72.0%)	0.072	2,205 (73.0%)	81 (69.2%)	0.429
Valve disease, *n* (%)	1,112 (45.4%)	40 (53.3%)	0.211	1,142 (37.8%)	67 (57.3%)	<0.001
Heart failure, *n* (%)	916 (37.4%)	47 (62.7%)	<0.001	300 (9.9%)	27 (23.1%)	<0.001
AF, *n* (%)	1,152 (47.0%)	39 (52.0%)	0.459	349 (11.6%)	27 (23.1%)	<0.001
CKD, *n* (%)	295 (12.0%)	15 (20.0%)	0.058	174 (5.8%)	13 (11.1%)	0.028
BUN, mg/dl	19.0 (14.0, 25.0)	27.0 (19.0, 42.0)	<0.001	18.0 (14.0, 24.0)	24.0 (16.0, 34.0)	<0.001
Serum albumin, g/dl	3.7 (3.2, 4.1)	3.20 (2.6, 3.6)	<0.001	3.4 (2.9, 3.7)	2.9 (2.4, 3.3)	<0.001
BAR	5.1 (3.9, 7.2)	10.0 (5.9, 13.5)	<0.001	5.3 (4.0, 7.6)	9.0 (5.4, 12.4)	<0.001
WBC, k/ul	8.4 (6.8, 11.4)	9.8 (8.0, 13.3)	0.007	9.5 (7.3, 13.8)	10.3 (6.9, 15.3)	0.338
PLT, k/u	210 (167, 258)	213 (169, 263)	0.874	177 (135, 224)	152 (104, 211)	<0.001
Hemoglobin, g/dl	12.2 (10.6, 13.6)	11.1 (9.4, 12.3)	<0.001	11.9 (10.2, 13.5)	10.4 (9.2, 11.9)	<0.001
SCr, mg/dl	1.0 (0.8, 1.3)	1.3 (1.1, 1.8)	<0.001	1.00 (0.8, 1.3)	1.34 (1.0, 2.0)	<0.001
Sodium, mEq/l	139 (137, 141)	138 (136, 140)	0.010	139 (136, 141)	138 (135, 141)	0.455
Potassium, mEq/l	4.10 (3.80, 4.40)	4.20 (3.80, 4.65)	0.204	4.0 (3.8, 4.4)	4.1 (3.7, 4.6)	0.439
Glucose, mg/dl	119 (100, 154)	123 (105, 169)	0.158	124 (103, 156)	140 (106, 165)	0.052
Anion gap	13.0 (12.0, 15.0)	15.00 (13.0, 16.5)	<0.001	9.0 (7.0, 12.0)	11.3 (8.4, 15.0)	<0.001
Bicarbonate	26.0 (24.0, 28.0)	23.0 (20.0, 26.5)	<0.001	25.0 (23.0, 27.0)	23.0 (21.0, 26.0)	<0.001
Chloride	104 (101, 106)	104 (100, 107)	0.826	105 (102, 108)	105 (101, 109)	0.412
ALT	22 (16.0, 33.0)	29 (19.0, 72.0)	<0.001	22.0 (15.0, 35.0)	36.0 (22.0, 81.8)	<0.001
AST	26.0 (20.0, 41.0)	53.0 (23.8, 124.3)	<0.001	28.0 (20.0, 47.0)	69.0 (32.0, 185.0)	<0.001
SOFA score	5.0 (3.0, 7.0)	6.0 (3.5, 10.0)	<0.001	5.0 (3.0, 7.0)	8.0 (5.0, 11.0)	<0.001
Vasoactive, *n* (%)	529 (21.6%)	51 (68.0%)	<0.001	1,063 (35.2%)	72 (61.5%)	<0.001

*MIMIC, Medical Information Mart for Intensive Care; BMI, body mass index; SBP, systolic blood pressure; DBP, diastolic blood pressure; CHD, coronary heart disease; AF, atrial fibrillation; CKD, chronic kidney disease; BUN, blood urea nitrogen; BAR, blood urea nitrogen to serum albumin ratio; WBC, white blood cell count; PLT, platelets; SCr, serum creatine; ALT, alanine aminotransferase; AST, aspartate aminotransferase; SOFA, sequential organ failure assessment; ICU, intensive care units*.

### Relationship Between BAR and Outcomes

**Figure 1:**
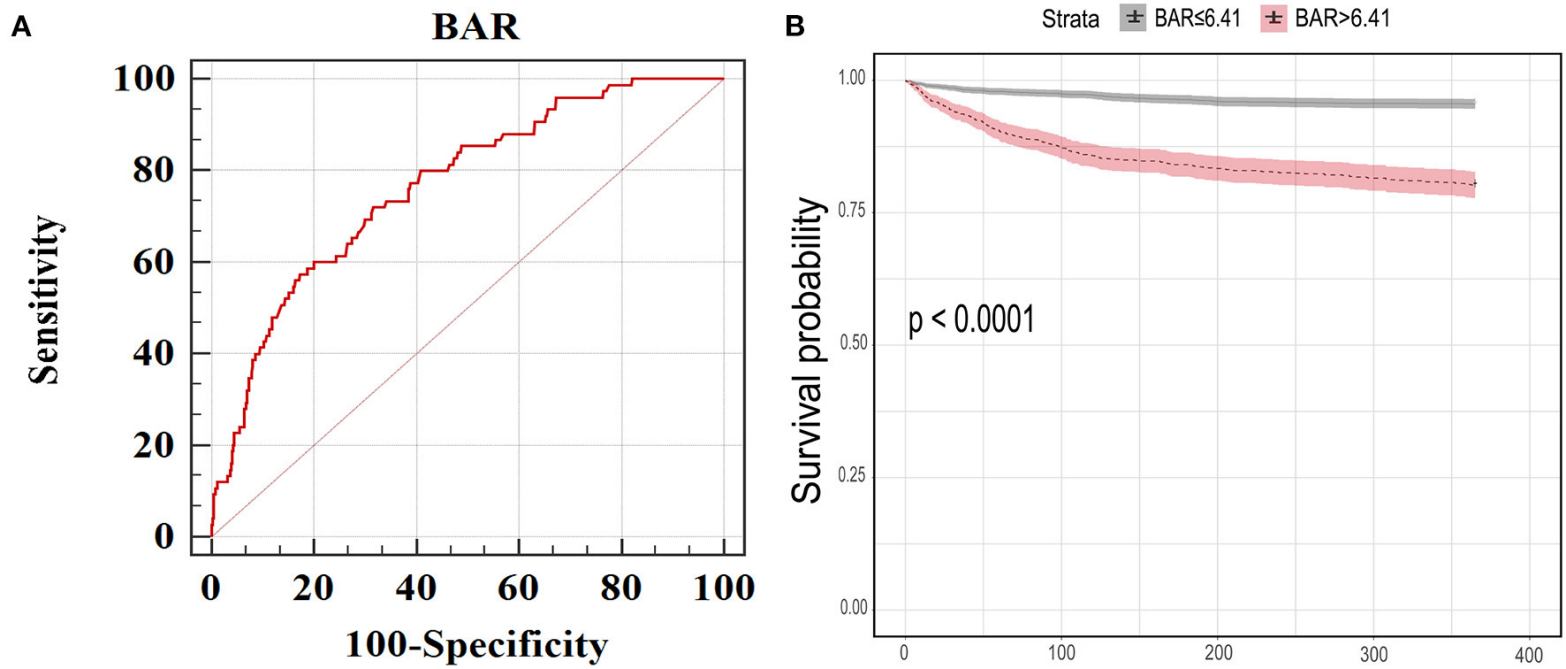
**(A)** The receiver operating characteristic curve of BAR for predicting in-hospital mortality in MIMIC III. The best cut-off value was >6.41 (mg/g). Area under the curve was 0.766 and the sensitivity, specificity, 95% CI lower and 95% CI upper were 72.0, 68.5%, 0.749 and 0.783, respectively. **(B)** Kaplan Meier curves for 1-year mortality stratified by high and low BAR in MIMIC III dataset. BAR, blood urea nitrogen to serum albumin ratio; 95% CI, 95% confidence interval; MIMIC, Medical Information Mart for Intensive Care.

According to ROC curve analysis, the optimal cutoff value of BAR for predicting in-hospital mortality in the MIMIC III was > 6.41 mg/g (AUC, 0.766; 95% confidence interval [CI], 0.749–0.783; Figure 1A). This cutoff value was then used to classify the patients into a low (≤6.41) and high (> 6.41) BAR group. In the MIMIC III, patients with increased BAR levels showed significantly higher rate of in-hospital, ICU and 1-year deaths, and longer lengths of ICU and hospital stays compared to patients with decreased BAR levels (Table 2). Similar results were observed in the eICU. The incidence of primary and secondary endpoints was also higher in the elevated BAR group (Table 2). Kaplan–Meier curves illustrated the 1-year survival difference between the two BAR groups (Figure 1B). Subjects with high BAR levels had significantly higher 1-year mortality risk than those with lower BAR levels (log-rank *p* < 0.001).

**Table 2:** Clinical outcomes between study cohorts.

**Outcomes**	**Low BAR (≤6.41)**	**High BAR (>6.41)**	***P***
**MIMIC III**	***N*** **=** **1,700**	***N*** **=** **827**	
In-hospital mortality, *n* (%)	21 (1.2%)	54 (6.5%)	<0.001
ICU mortality, *n* (%)	19 (1.1%)	44 (5.3%)	<0.001
1-year mortality, *n* (%)	68 (4.0%)	170 (20.6%)	<0.001
ICU stay ≥ 5 days, *n* (%)	338 (19.9%)	390 (47.2%)	<0.001
Hospital stay ≥ 15 days, *n* (%)	274 (16.1%)	338 (40.9%)	<0.001
**eICU**	***N*** **=** **1,980**	***N*** **=** **1,158**	
In-hospital mortality, *n* (%)	38 (1.9%)	79 (6.8%)	<0.001
ICU mortality, *n* (%)	23 (1.2 %)	62 (5.4%)	<0.001
ICU stay ≥ 5 days, *n* (%)	431 (21.8%)	396 (34.2%)	<0.001
Hospital stay ≥ 15 days, *n* (%)	266 (13.4%)	374 (32.3%)	<0.001

*MIMIC, Medical Information Mart for Intensive Care; BAR, blood urea nitrogen to serum albumin ratio; ICU, intensive care units*.

Multivariate regression analyses were utilized to evaluate the significance of BAR in predicting unfavorable outcomes. In the MIMIC III, despite adjustment for other confounding variables a 1-point increase of BAR was remarkably correlated with higher risk of in-hospital, ICU and 1-year death, and prolonged ICU (≥5 days) and hospital stays (≥15 days) (OR [95% CI]:1.07 [1.02–1.12], *p* = 0.003 for In-hospital death; OR [95% CI]:1.07 [1.02–1.13], *p* = 0.006 for ICU death; HR [95% CI]: 1.06 [1.03, 1.08], *p* < 0.001 for 1-year death; OR [95% CI]: 1.08 [1.05–1.11], *p* < 0.001 for ICU stay ≥5 days; and OR [95% CI]: 1.08 [1.05–1.11], *p* < 0.001 for hospital stay ≥15 days, Table 3). High BAR level also retained its independent predictive value of the primary and secondary outcomes even after adjusting for risk factors (all *p* < 0.05, Table 3). The aforementioned results were successfully validated in the eICU. Multivariate regression analyses also showed that higher BAR level was independently associated with primary and secondary endpoints, irrespective of BAR being a nominal or continuous variable (all *p* < 0.05, Table 3).

**Table 3:** Predictive value of BAR for adverse endpoints.

	**Unadjusted**		**Adjusted**	
	**OR/HR (95%Cl)**	***P***	**OR/HR (95%Cl)**	***P***
**MIMIC III ^#^**				
**BAR as continuous variable^a^**				
In-hospital mortality	1.12 (1.09–1.15)	<0.001	1.07 (1.02–1.12)	0.003
ICU mortality	1.11 (1.08–1.14)	<0.001	1.07 (1.02–1.13)	0.006
1-year mortality	1.09 (1.08–1.10)	<0.001	1.06 (1.03–1.08)	<0.001
ICU stay ≥ 5 days	1.15 (1.13–1.18)	<0.001	1.08 (1.05–1.11)	<0.001
Hospital stay ≥ 15 days	1.14 (1.11–1.16)	<0.001	1.08 (1.05–1.11)	<0.001
**BAR as nominal variable^b^**				
In-hospital mortality	5.59 (3.35–9.31)	<0.001	2.70 (1.47–4.96)	0.001
ICU mortality	4.97 (2.88–8.57)	<0.001	2.77 (1.44–5.32)	0.002
1-year mortality	5.61 (4.24–7.43)	<0.001	2.73 (1.97–3.79)	<0.001
ICU stay ≥ 5 days	3.60 (3.00–4.31)	<0.001	1.90 (1.50–2.41)	<0.001
Hospital stay ≥ 15 days	3.60 (2.98–4.35)	<0.001	2.06 (1.61–2.63)	<0.001
**eICU^#^**				
**BAR as continuous variable^a^**				
In-hospital mortality	1.10 (1.08–1.13)	<0.001	1.06 (1.01–1.10)	0.013
ICU mortality	1.10 (1.08–1.13)	<0.001	1.06 (1.02–1.11)	0.006
ICU stay ≥ 5 days	1.06 (1.04–1.07)	<0.001	1.03 (1.01–1.06)	0.018
Hospital stay ≥ 15 days	1.13 (1.10–1.15)	<0.001	1.08 (1.05–1.11)	<0.001
**BAR as nominal variable^b^**				
In-hospital mortality	3.74 (2.52–5.55)	<0.001	2.21 (1.17–4.20)	0.015
ICU mortality	4.81 (2.97–7.81)	<0.001	2.42 (1.19–4.92)	0.015
ICU stay ≥ 5 days	1.87 (1.59–2.20)	<0.001	1.47 (1.14–1.89)	0.003
Hospital stay ≥ 15 days	3.07 (2.57–3.67)	<0.001	2.04 (1.57–2.65)	<0.001

a *The OR was examined by per 1-point increase of BAR*.

b *The OR was examined regarding the low BAR as reference*.

# *The baseline model includes variables that are significant in univariate logistic proportional hazard analysis in MIMIC III, including age, SBP, heart rate, hypertension, heart failure, CKD, WBC, hemoglobin, SCr, sodium, glucose, anion gap, bicarbonate, ALT, AST, SOFA score and vasoactive (details shown in Supplementary Table 2)*.

*OR, odds ratio; HR, hazard ratio; 95% CI, 95% confidence interval; MIMIC, Medical Information Mart for Intensive Care; BAR, blood urea nitrogen to serum albumin ratio; ICU, intensive care units*.

### Subgroup Analyses

**Figure 2:**
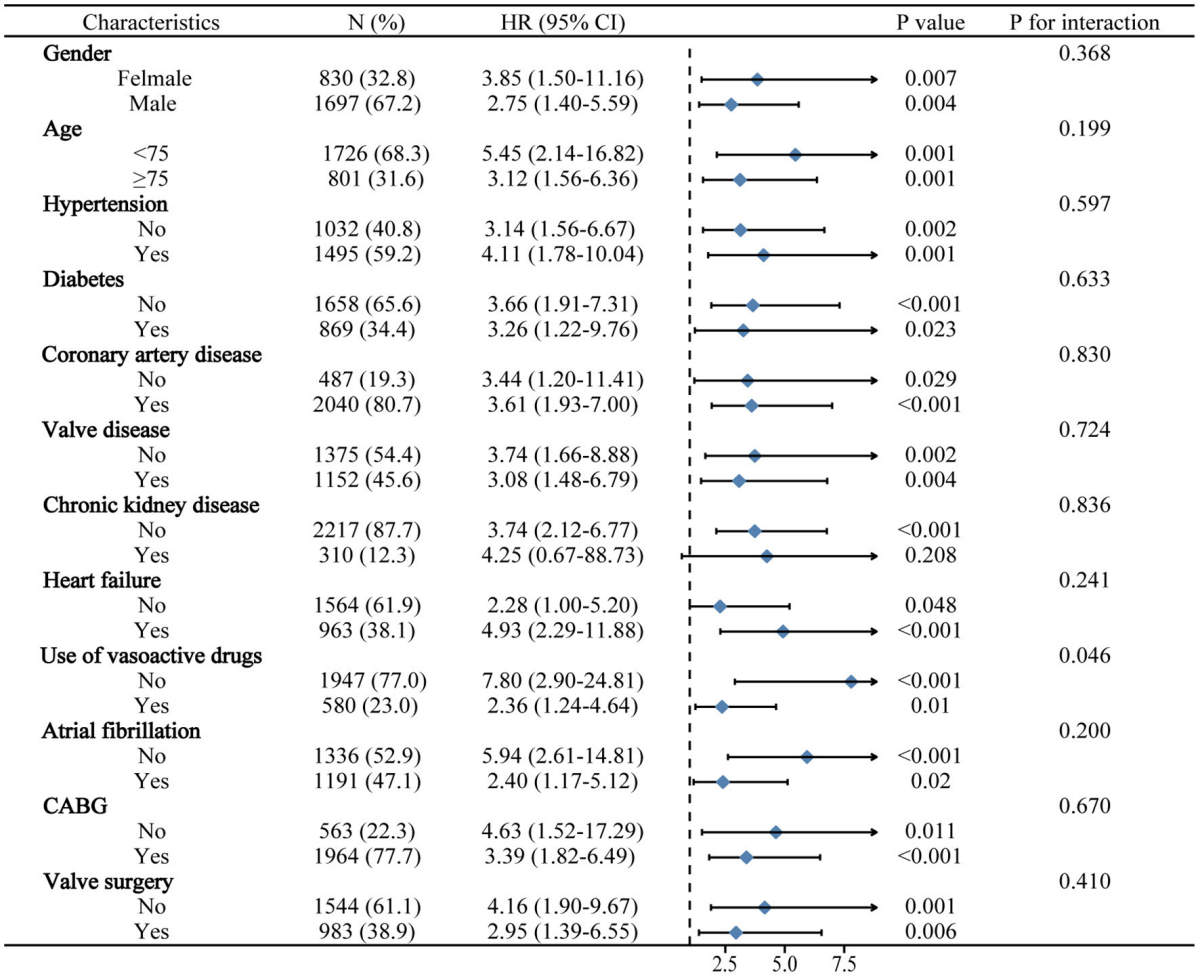
Logistic regression analysis evaluating prognostic value of BAR in various subgroups in MIMIC III. The OR was examined regarding the low BAR as reference. BAR, blood urea nitrogen to serum albumin ratio; OR, odds ratio; 95% CI, 95% confidence interval; MIMIC, Medical Information Mart for Intensive Care.

The significance of BAR for predicting in-hospital mortality was further evaluated in different subclasses in MIMIC III (Figure 2). Elevated BAR was consistently correlated with a higher risk of in-hospital mortality in all subgroups, except for the subgroup of patients with CKD (*p* = 0.208). Remarkably, the predictive significance of BAR appeared to be more prominent in patients who did not vasoactive drugs (*P*_interaction_ = 0.046).

### Incremental Significance of BAR in Predicting In-hospital Death

**Figure 3:**
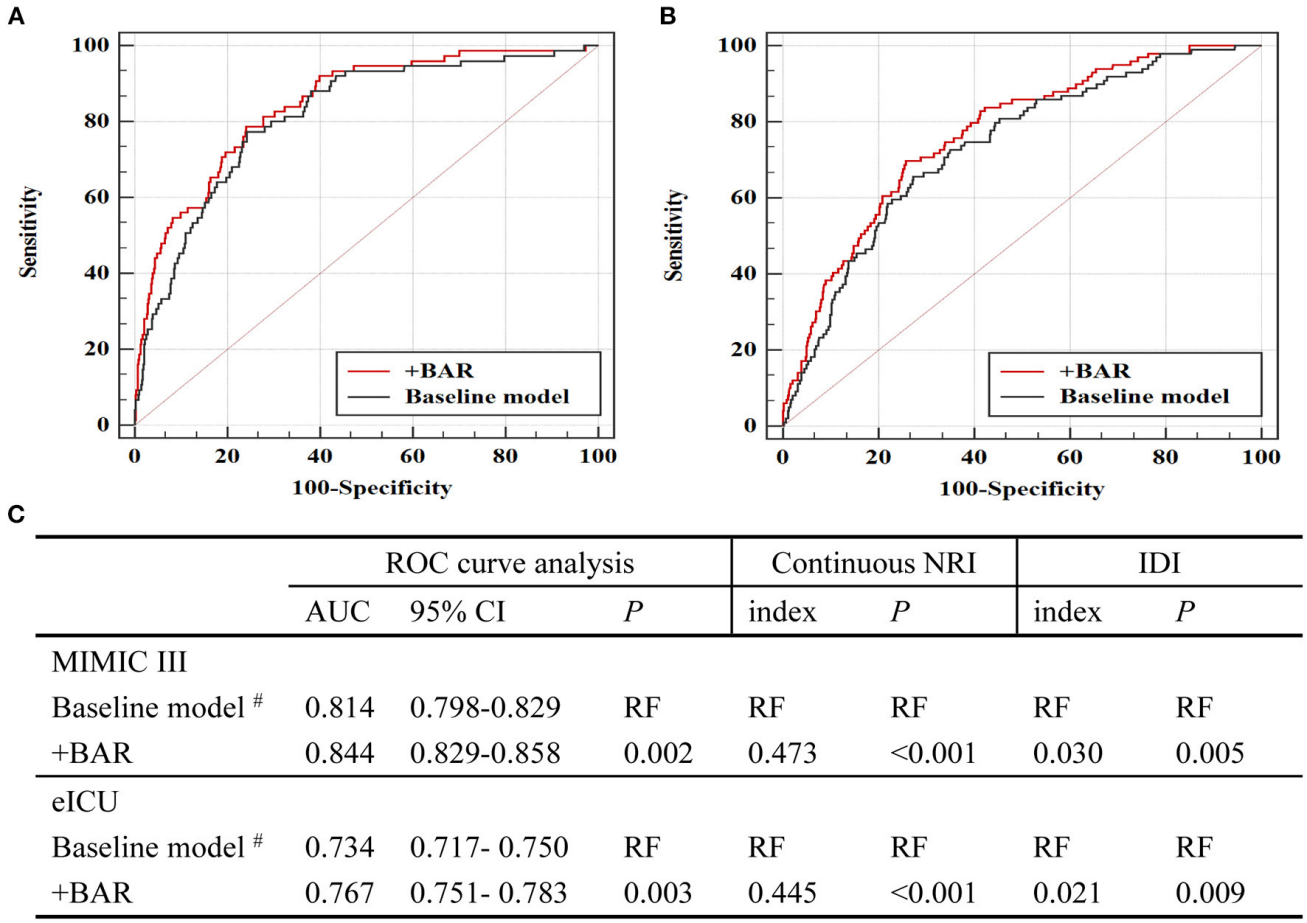
Evaluation of predictive models for in-hospital mortality. **(A)** ROC curve evaluating predictive value of various models for in-hospital mortality in MIMIC III. **(B)** ROC curve evaluating predictive value of various models for in-hospital mortality in eICU. **(C)** C-statistics for discrimination ability of various models. ROC, receiver operator characteristic; AUC, area under the curve; 95% CI, 95% confidence interval; BAR, blood urea nitrogen to serum albumin ratio; MIMIC, Medical Information Mart for Intensive Care; ICU, intensive care units. ^#^The baseline model includes variables that are significant in multivariate logistic proportional hazard analysis in MIMIC III, including age, SBP, hypertension, CKD, hemoglobin and vasoactive.

By adding BAR to a baseline model that contained factors that were significant in multivariate logistic regression analysis, the AUCs for predicting in-hospital mortality increased significantly from 0.814 to 0.844 (*P* = 0.002, Figures 3A,C). Additionally, the continuous NRI and IDI showed significant improvement after adding BAR to the baseline model (*p* < 0.05, Figure 3C). The validation cohort (eICU) further confirmed incremental prognostic value of BAR for predicting in-hospital mortality. When BAR was added to the baseline risk model, we also observed a significant additional improvement in AUCs, continuous NRI and IDI (all *p* < 0.05, Figures 3B,C).

## Discussion

The main findings of this paper were that BAR, as a categorical or continuous variable, was significantly related to poor prognosis of cardiac surgery patients persists even after adjusting for confounding variables, and BAR could provide additional prognostic information for in-hospital mortality beyond the baseline model. Consistent results were obtained in the external validation cohort.

High BAR values resulted from an elevated BUN or low albumin levels. Studies have reported that both elevated BUN and hypoalbuminemia were strongly related to poor prognoses in patients with cardiovascular diseases including ACS, AMI, HF and ischemic stroke (7–9, 20–25), which indicated the possibility of using BUN or albumin for risk stratification in patients with cardiovascular disease. Previous findings have also demonstrated that elevated BUN or decreased albumin were correlated with increased risk of mortality in ICU patients (26, 27). Moreover, the prognostic implication of preoperative hypoalbuminemia has also been verified in patients undergoing major vascular surgery (12, 28) or valve surgeries (10). However, whether a combination of BUN and albumin could predict adverse outcomes of cardiac surgery patients has not been documented. Findings from this present study showed that BAR was a predictor of poor outcomes in patients undergoing cardiac surgery, which was further confirmed by external validation, suggesting the prognostic reliability of BAR.

Studies have shown that the level of BUN and albumin are influenced by age and that the results should be cautiously interpreted, especially for elderly patients (29, 30). Our subgroup analysis presented that the association between BAR and in-hospital death persisted in patients of different ages (<75 or ≥75 years). In multivariate analysis, BAR was consistently related to adverse outcomes even after correcting for confounding factors such as Scr and CKD, suggesting its prognostic significance was independent of renal function. However, subgroup analysis suggested that the correlation between high BAR and in-hospital death became non-significant in patients with CKD. But, the interaction was not significant according with or without CKD. We speculated that this observation could be related to the few cases with CKD in the investigated cohort.

The concentration of BUN can be affected by various factors such as protein intake and consumption, blood volume status, urea excretion, and reabsorption in the renal tubules. The level of albumin can be associated with liver synthesis, catabolism, and extravasation from vessels, and more. The BAR can reduce these mentioned influencing factors and better integrate the clinical implication of BUN and albumin. The underlying mechanisms that may account for the association between BAR and prognosis may be attributed to the following reasons. Generally, patients requiring cardiac surgery are often accompanied by a low-perfusion state, resulting in the activation of the sympathetic nervous system (SNS) and RAAS, and the release of arginine vasopressin (AVP). The activation of RASS and SNS are associated with increased water and sodium absorption, leading to increased passive reabsorption of urea in the renal tubules (13, 14). Besides, the release of AVP can lead to the excessive upregulation of urea transporters in the intramedullary collecting duct, thus promoting the reabsorption of urea (14, 31). The relevance between hypoalbuminemia and cardiac surgery may be interpreted by several reasons. Hypoproteinemia is an important marker of malnutrition. The prognostic significance of malnutrition has also been identified in the risk stratification of patients undergoing cardiac surgery (32, 33). Since all patients in this study underwent surgery, they could have experienced a certain extent of appetite loss and disease-related inflammatory conditions leading to a decrease in albumin levels. Additionally, another mechanism to consider could be serum albumin aggravated oxidative stress and inflammatory response in cardiac surgery. Serum albumin is considered as a significant circulating antioxidant (15). Numerous studies have shown that oxidative stress was related to an increased risk of complications after cardiac surgery (34, 35). Another potential property of serum albumin is its anti-inflammatory effect (16). It was established that inflammation has a major part in the progression and prognosis of cardiac surgery (34, 36).

Our study is subject to some limitations. First, we did not evaluate the dynamic changes of BAR. Changes in BAR during hospitalization could provide better prognostic information. Second, both the MIMIC III and eICU databases did not contain data on EuroSCORE II; thus, an association between BAR and EuroSCORE II could not be determined. Third, long-term follow-up data were not recorded in the eICU database, and we could not validate the relationship between BAR and 1-year mortality. Lastly, the retrospective nature of this study could have inevitably contained a certain level of selection bias.

## Conclusion

Elevated preoperative BAR was found to be a prominent risk predictor of unfavorable outcomes in cardiac surgery patients.

## Data Availability Statement

Publicly available datasets were analyzed in this study. This data can be found at: The MIMIC III and eICU databases are publicly available from https://mimic.mit.edu/ and https://eicu-crd.mit.edu/.

## Author Contributions

LY was responsible for designing protocol, conducting the search, extracting and analyzing data from MIMIC-III and eICU, interpreting results, and creating summary of findings tables. HS and XW was responsible for designing the review protocol and extracting data. QD and PG contributed to updating reference lists and provided feedback on the report. YS contributed to analyzing data, interpreting results, as well as creating tables and figures, and writing paper. All authors contributed to the article and approved the submitted version.

## Conflict of Interest

The authors declare that the research was conducted in the absence of any commercial or financial relationships that could be construed as a potential conflict of interest.

## Publisher's Note

All claims expressed in this article are solely those of the authors and do not necessarily represent those of their affiliated organizations, or those of the publisher, the editors and the reviewers. Any product that may be evaluated in this article, or claim that may be made by its manufacturer, is not guaranteed or endorsed by the publisher.
