# Supplementary material for: Elevated Blood Urea Nitrogen to Serum Albumin Ratio Is an Adverse Prognostic Predictor for Patients Undergoing Cardiac Surgery
Source: Front Cardiovasc Med. 2022 May 4;9:888736. doi: 10.3389/fcvm.2022.888736 (PMC9114352; doi:10.3389/fcvm.2022.888736)
Supplement: Supplementary file 1 [file Table_1.doc]

**Supplementary table 1** Characteristics of the study population.

| Variables | MIMIC-III | eICU | *P* |
| --- | --- | --- | --- |
|  | N=2527 | N=3138 |  |
| Age, years | 68.9 (60.1, 77.1) | 68.0 (60.0, 76.0) | 0.026 |
| Male, n (%) | 1697 (67.2%) | 2157 (68.7%) | 0.214 |
| BMI, kg/m2 | 27.9 (24.6, 31.7) | 28.9 (25.3, 33.2) | < 0.001 |
| SBP, mmHg | 115.0 (104.0, 129.0) | 120.0 (105.0, 137.0) | <0.001 |
| DBP, mmHg | 59.0 (52.0, 67.0) | 65.0 (56.0,76.0) | < 0.001 |
| Heart rate, bpm | 84.0 (77.0, 90.0) | 80.0 (70.0, 91.0) | < 0.001 |
| Hypertension, n (%) | 1495 (59.2%) | 651 (20.7%) | < 0.001 |
| Diabetes, n (%) | 869 (34.4%) | 547 (17.4%) | < 0.001 |
| CHD, n (%) | 2040 (80.7%) | 2286 (72.8%) | < 0.001 |
| Valve disease, n (%) | 1152 (45.6%) | 1209 (38.5%) | < 0.001 |
| Heart failure, n (%) | 963 (38.1%) | 327 (10.4%) | < 0.001 |
| AF, n (%) | 1191 (47.1%) | 376 (12.0%) | < 0.001 |
| CKD, n (%) | 310 (12.3%) | 187 (6.0%) | < 0.001 |
| BUN, mg/dl | 19.0 (15.0, 25.0) | 18.0 (14.0, 25.0) | < 0.001 |
| Serum albumin, g/dl | 3.7 (3.2, 4.0) | 3.3 (2.9, 3.7) | < 0.001 |
| BAR | 5.2 (3.9, 7.4) | 5.4 (4.1, 7.8) | 0.005 |
| WBC, k/ul | 8.5 (6.8, 11.5) | 9.6 (7.3, 13.9) | <0.001 |
| PLT, k/u | 210.0 (167.0, 258.0) | 177.0 (133.0, 223.0) | <0.001 |
| Hemoglobin, g/dl | 12.2 (10.6, 13.6) | 11.9 (10.1, 13.5) | <0.001 |
| SCr, mg/dl | 1.0 (0.8, 1.3) | 1.0 (0.8, 1.3) | 0.979 |
| Sodium, mEq/l | 139.0 (137.0, 141.0) | 139.0 (136.0, 141.0) | 0.001 |
| Potassium, mEq/l | 4.1 (3.8, 4.4) | 4.0 (3.8, 4.4) | <0.001 |
| Glucose, mg/dl | 119.0 (100.0, 155.0) | 124.0 (103.0, 156.0) | 0.001 |
| Anion gap | 13.0 (12.0, 15.0) | 9.0 (7.0, 12.0) | < 0.001 |
| Bicarbonate | 26.0 (24.0, 28.0) | 25.0 (23.0, 27.0) | <0.001 |
| Chloride | 104.0 (101.0, 106.0) | 105.0 (102.0, 108.0) | < 0.001 |
| ALT | 22.0 (16.0, 34.0) | 23.0 (15.0, 36.0) | 0.620 |
| AST | 26.0 (20.0, 42.0) | 29.0 (20.0, 49.0) | 0.001 |
| SOFA score | 5 (3, 7) | 5 (3, 7) | <0.001 |
| Vasoactive, n (%) | 580 (23.0%) | 1135 (36.2%) | < 0.001 |
| surgery |  |  | < 0.001 |
| CABG, n (%) | 1544 (61.1%) | 1889 (60.2%) |  |
| Valve surgery, n (%) | 563 (22.3%) | 885 (28.2%) |  |
| CABG + valve surgery, n (%) | 420 (16.6%) | 364 (11.6%) |  |
| In-hospital mortality, n (%) | 75 (3.0%) | 117 (3.7%) | 0.134 |
| ICU mortality, n (%) | 63 (2.5%) | 85 (2.7%) | 0.673 |
| 1-year mortality, n (%) | 238 (9.4%) | -- | -- |
| Length of ICU stay, days | 3.0 (1.4, 5.4) | 2.9 (1.6, 5.1) | 0.013 |
| Length of hospital stay, days | 10.0 (7.2, 14.8) | 9.1 (6.3, 13.7) | <0.001 |

MIMIC, Medical Information Mart for Intensive Care; BMI, body mass index; SBP, systolic blood pressure; DBP, diastolic blood pressure; CHD, coronary heart disease; AF, atrial fibrillation; CKD, chronic kidney disease; BUN, blood urea nitrogen; BAR, blood urea nitrogen to serum albumin ratio; WBC, white blood cell count; PLT, platelets; SCr, serum creatine; ALT, alanine aminotransferase; AST, aspartate aminotransferase; SOFA, sequential organ failure assessment; CABG, coronary artery bypass graft surgery; ICU, intensive care units.

**Supplementary table 2** Univariate and multivariate regression analyses for in-hospital mortality in MIMIC III.

|  | Unadjusted | | Adjusted | |
| --- | --- | --- | --- | --- |
|  | OR (95%CI) | *P* | OR (95%CI) | *P* |
| Age, years | 1.02 (1.00-1.04) | 0.024 | 1.02 (1.00-1.05) | 0.039 |
| Male, n (%) | 0.92 (0.57-1.49) | 0.733 |  |  |
| BMI, kg/m^2^ | 1.03 (0.99-1.07) | 0.070 |  |  |
| SBP, mmHg | 0.98 (0.97-0.99) | 0.005 | 0.98 (0.97-0.99) | 0.010 |
| DBP, mmHg | 0.97 (0.95-0.99) | 0.001 |  |  |
| Heart rate, bpm | 1.03 (1.01-1.05) | <0.001 | 1.01 (0.99-1.03) | 0.307 |
| Hypertension, n (%) | 0.36 (0.22-0.58) | <0.001 | 0.44 (0.25-0.79) | 0.005 |
| Diabetes, n (%) | 0.95 (0.59-1.55) | 0.845 |  |  |
| CHD, n (%) | 0.60 (0.36-1.01) | 0.054 |  |  |
| Valve disease, n (%) | 1.38 (0.87-2.18) | 0.173 |  |  |
| Heart failure, n (%) | 2.81 (1.75-4.53) | <0.001 | 1.01 (0.58-1.76) | 0.970 |
| AF, n (%) | 1.22 (0.77-1.94) | 0.392 |  |  |
| CKD, n (%) | 1.83 (1.02-3.26) | 0.041 | 0.46 (0.21-0.99) | 0.046 |
| BUN, mg/dl | 1.03 (1.02-1.04) | <0.001 |  |  |
| Serum albumin, g/dl | 0.32 (0.23-0.44) | <0.001 |  |  |
| BAR | 1.12 (1.09-1.15) | <0.001 | 1.07 (1.02-1.12) | 0.003 |
| WBC, k/ul | 1.04 (1.00-1.07) | 0.047 | 0.96 (0.90-1.01) | 0.121 |
| PLT, k/u | 1.00 (0.99-1.00) | 0.981 |  |  |
| Hemoglobin, g/dl | 0.78 (0.71-0.87) | <0.001 | 0.86 (0.75-0.98) | 0.027 |
| SCr, mg/dl | 1.28 ( 1.16-1.41) | <0.001 | 1.11 (0.93-1.34) | 0.255 |
| Sodium, mEq/l | 0.91 (0.85-0.98) | 0.011 | 0.98 (0.91-1.06) | 0.683 |
| Potassium, mEq/l | 1.32 (0.90-1.93) | 0.153 |  |  |
| Glucose, mg/dl | 1.00 (1.00-1.01) | 0.010 | 1.00 (0.99-1.00) | 0.412 |
| Anion gap | 1.21 (1.13- 1.30) | <0.001 | 1.04 (0.94-1.15) | 0.457 |
| Bicarbonate | 0.85 (0.80-0.91) | <0.001 | 0.95 (0.88-1.03) | 0.181 |
| Chloride | 1.00 (0.95-1.06) | 0.899 |  |  |
| ALT | 1.00 (1.00-1.00) | 0.013 | 1.00 (0.99-1.00) | 0.607 |
| AST | 1.00 (1.00-1.00) | 0.009 | 1.00 (0.99-1.00) | 0.418 |
| SOFA score | 1.21 (1.12-1.30) | <0.001 | 0.97 (0.89-1.06) | 0.546 |
| Vasoactive, n (%) | 7.72 (4.71-12.67) | <0.001 | 5.83 (3.25-10.46) | <0.001 |

MIMIC, Medical Information Mart for Intensive Care; BMI, body mass index; SBP, systolic blood pressure; DBP, diastolic blood pressure; CHD, coronary heart disease; AF, atrial fibrillation; CKD, chronic kidney disease; BUN, blood urea nitrogen; BAR, blood urea nitrogen to serum albumin ratio; WBC, white blood cell count; PLT, platelets; SCr, serum creatine; ALT, alanine aminotransferase; AST, aspartate aminotransferase; SOFA, sequential organ failure assessment.
