# Supplementary material for: Elevated Blood Urea Nitrogen to Serum Albumin Ratio Is an Adverse Prognostic Predictor for Patients Undergoing Cardiac Surgery
Source: Front Cardiovasc Med. 2022 May 4;9:888736. doi: 10.3389/fcvm.2022.888736 (PMC9114352; doi:10.3389/fcvm.2022.888736)
Supplement: Supplementary file 2 [file Data_Sheet_1.doc]

**
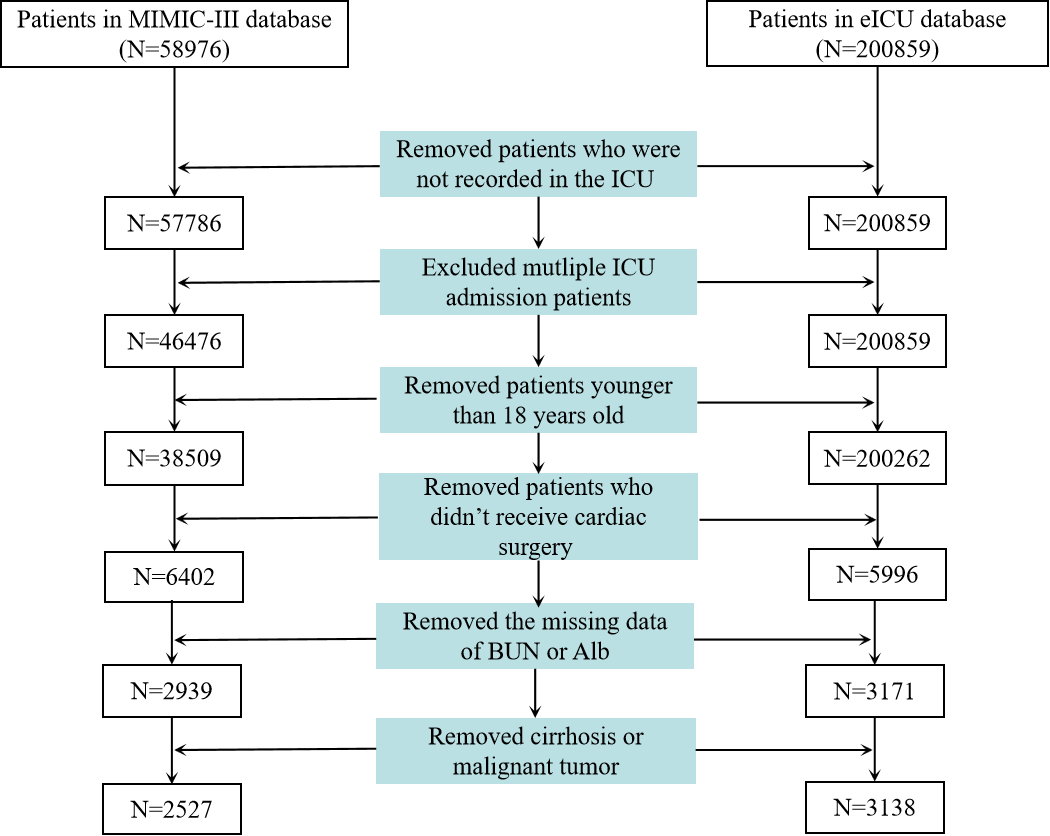
Supplementary Figure 1.** Flow chart of the study population enrollment**.**

MIMIC, Medical Information Mart for Intensive Care; ICU, intensive care unit; CABG, coronary artery bypass graft surgery;BUN, blood urea nitrogen; Alb, albumin.
